# Elevated vascular endothelial growth factor a is associated with disruption of default network connectivity in older adults

**DOI:** 10.1007/s11682-025-00969-z

**Published:** 2025-02-04

**Authors:** Arunima Kapoor, Jung Yun Jang, Allison C. Engstrom, Trevor Lohman, Shubir Dutt, John Paul M. Alitin, Isabel J. Sible, Anisa Marshall, Fatemah Shenasa, Aimee Gaubert, Amy Nguyen, David Robert Bradford, Kathleen Rodgers, S. Duke Han, Daniel A. Nation

**Affiliations:** 1 Department of Psychological Science, University of California, Irvine, Irvine, CA USA; 2 Institute for Memory Impairments and Neurological Disorders, University of California, Irvine, Irvine, CA USA; 3 Leonard Davis School of Gerontology, University of Southern California, 3715 McClintock Avenue, Los Angeles, CA 90089 USA; 4 Department of Neurology, Weill Institute for Neurosciences, Memory and Aging Center, University of California, San Francisco, San Francisco, CA USA; 5 Department of Psychology, University of Southern California, Los Angeles, CA USA; 6 Center for Innovations in Brain Science, Department of Pharmacology, University of Arizona, Tucson, AZ USA; 7 Department of Family Medicine, Department of Neurology, University of Southern California, Los Angeles, CA USA; 8 Keck School of Medicine, University of Southern California, Los Angeles, CA USA

**Keywords:** Vascular endothelial growth factor A, Default network connectivity

## Abstract

Vascular Endothelial Growth Factor A (VEGF-A) is an angiogenic signaling protein involved in the maintenance of the cerebral vasculature. No prior study has explored whether plasma VEGF-A levels may be associated with brain functional connectivity changes, such as disruption of the default mode network (DMN), which often precedes the development of cognitive changes in aging. Seventy-six independently living older adults (mean age = 70.3 years; SD = 7.5; 31.6% male) free of dementia or clinical stroke underwent venipuncture and brain MRI. Plasma was assayed for VEGF-A. Using resting state functional MRI, region of interest (ROI) to ROI connectivity and graph theory analysis were conducted to determine average connectivity and global efficiency between each of the following ROIs comprising the DMN: medial prefrontal cortex, lateral parietal cortex and precuneus cortex. Multiple linear regression analysis revealed a significant negative association between VEGF-A levels and DMN connectivity (B = − 0.14, 95% CI (-0.26, − 0.01), *p* =.038), accounting for age, sex, education, and vascular risk factors. Graph theory analysis similarly revealed that VEGF-A levels are associated with global efficiency of the entire network (B = − 0.18, *p* =.004). These findings suggest that VEGF-A may be elevated early in the progression of neurocognitive disorders. Whether higher levels of VEGF-A contribute to the pathogenesis of neurocognitive disorders or play a protective role in preserving cognitive function warrants further investigation. Clinical Trial Number: N/A; None.

**Supplementary Information:**

The online version contains supplementary material available at 10.1007/s11682-025-00969-z.

## Introduction

Vascular dysfunction is known to contribute to default mode network (DMN) connectivity and cognitive impairment (Qin et al., 2021). Vascular Endothelial Growth Factor A (VEGF-A) is an angiogenic signaling protein involved in the maintenance of the cerebral vasculature, including endothelial cell proliferation and migration (Lange et al., 2016). While these functions may be neuroprotective, VEGF-A also regulates microvascular density and acts as a vascular permeability factor, which triggers leaky blood vessel formation and breakdown of the blood brain barrier (Dvorak et al., 1995). Therefore, whether elevated VEGF-A levels indicate a pathophysiological response or protective mechanism (Holmes & Zachary, 2005; Podar & Anderson, 2005; Weis & Cheresh, 2005), and whether VEGF-A levels affect cognitive functioning, remains unclear.

Studies examining VEGF-A production and response in older adults have shown reduced mRNA and protein levels with increasing age (Lähteenvuo & Rosenzweig, 2012). Furthermore, cognitive decline in aging and Alzheimer’s disease and related dementias is often preceded by changes in functional connectivity among brain regions, particularly disruption of the default mode network (Hedden et al., 2009; Köbe et al., 2021). Prior studies have examined DMN connectivity and vascular dysfunction in disease states; however, few studies have examined whether vascular dysfunction may be associated with DMN connectivity in older adults without dementia. To date, no prior study has explored whether VEGF-A levels may be associated with changes in DMN connectivity prior to the development of cognitive decline.

Examining the association between VEGF-A and DMN connectivity may elucidate whether angiogenic dysregulation is associated with early brain functional network connectivity changes. In a community-based sample of older adults without dementia, we hypothesized that VEGF-A levels may be associated with DMN connectivity, implicating involvement of VEGF-A in functional connectivity changes that precede cognitive decline.

## Methods

### Participants

All participants were recruited from the community, and all study procedures were conducted as part of the Vascular Senescence and Cognition (VaSC) Study at the University of Southern California (USC) and University of California, Irvine (UCI). Older adults aged 55 years or older who were living independently were included. Exclusion criteria were a history of clinical stroke, dementia, major neurological or psychiatric disorder impacting cognition, MRI contraindication, current organ failure and other systemic or neurological illness that may impact central nervous system function. All participants were free of dementia, which was established based on independence in daily living, Clinical Dementia Rating score of 0 or 0.5 and neuropsychological testing to screen for absence of major neurocognitive disorder. History of vascular risk factors was determined by clinical interview. Participants with incidental findings on brain magnetic resonance imaging (MRI) and history of cardiovascular disease were excluded from the current analysis, given that these factors may unduly influence the levels of circulating VEGF-A. This study was conducted in accordance with the Declaration of Helsinki and approved by the Institutional Review Board at UCI and USC; all participants gave informed consent and underwent brain MRI and venipuncture. The data that support the findings of this study are available from the corresponding author upon request.

### Neuroimaging

Participants underwent brain MRI on a 3T scanner (Siemens MAGNETOM Prisma System). The following sequences were examined for the current analysis: 3D T1-weighted MPRAGE anatomical scan for qualitative assessment of brain structures and abnormalities and resting state fMRI (rsfMRI) to determine blood-oxygen-level-dependent (BOLD) signal. Scan parameters are described in the Supplementary Material.

### Resting state fMRI analysis

BOLD scans were preprocessing using the default pipeline using the CONN toolbox, which has been described in detail elsewhere (Jang et al., 2021). Region-of-interest (ROI) to ROI connectivity was determined within the DMN based on average connectivity (Fisher’s z-transformed correlation coefficients) between the following pairs of ROIs (MNI coordinates): medial prefrontal cortex (1,55,-3), lateral parietal cortex (-39,-77,33 & 47,-67,29), and precuneus cortex (1,-61,38). We further conducted graph theory analysis among the same DMN ROIs as network nodes in CONN.

### Plasma VEGF-A levels

Venipuncture was performed after an overnight fast. Plasma was isolated by density gradient centrifugation. VEGF-A level was determined using the Meso Scale Discovery V-PLEX Human Biomarker 40-Plex Kit Angiogenesis Panel 1, following manufacturer’s protocol without modification and has been previously described in detail (Kapoor et al., 2021). VEGF-A values were log-transformed. *APOE* genotyping was conducted on the blood cell pellet fraction obtained from plasma separation, as previously described (Sible et al., 2022).

### General cognitive status

Participants underwent brief cognitive testing using the Dementia Rating Scale (DRS), which entails assessment of attention, initiation/perseveration (I/P), construction, conceptualization and memory. Age-adjusted Mayo’s Older Americans Normative Studies (MOANS) scaled score on DRS were further adjusted for sex and education and utilized for the current analysis.

### Statistical analyses

All analyses were performed using R Version 3.6.1 and IBM SPSS Statistics 28. The relationship between VEGF-A (independent predictor) and DMN average connectivity (dependent outcome) was examined using linear regression, adjusting for age, sex, and education. Given that vascular risk factors (defined as hypertension, dyslipidemia, diabetes, smoking, transient ischemic attack, or atrial fibrillation in this study) are known to affect functional connectivity (Köbe et al., 2021), analyses also adjusted for number of vascular risk factors (0–1 vs. ≥ 2). The association between DMN average connectivity and general cognitive status based on the DRS was examined using linear regression. Significance threshold was set at *p* <.05. For graph theory analysis, we utilized a 0.15 correlation threshold for the network edges to examine global efficiency (GE) with threshold of p-FDR < 0.05.

## Results

**Fig. 1:**
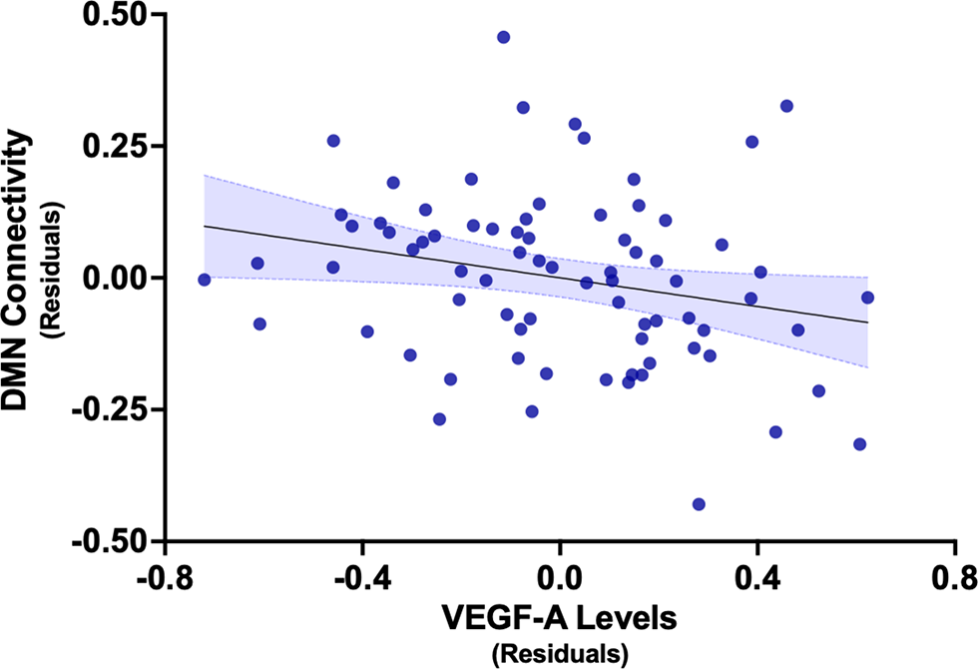
Partial regression plot of association between VEGF-A levels and DMN connectivity. Note: Higher VEGF-A levels are associated with decreased connectivity, adjusting for age, sex, education, and vascular risk factors

**Fig. 2:**
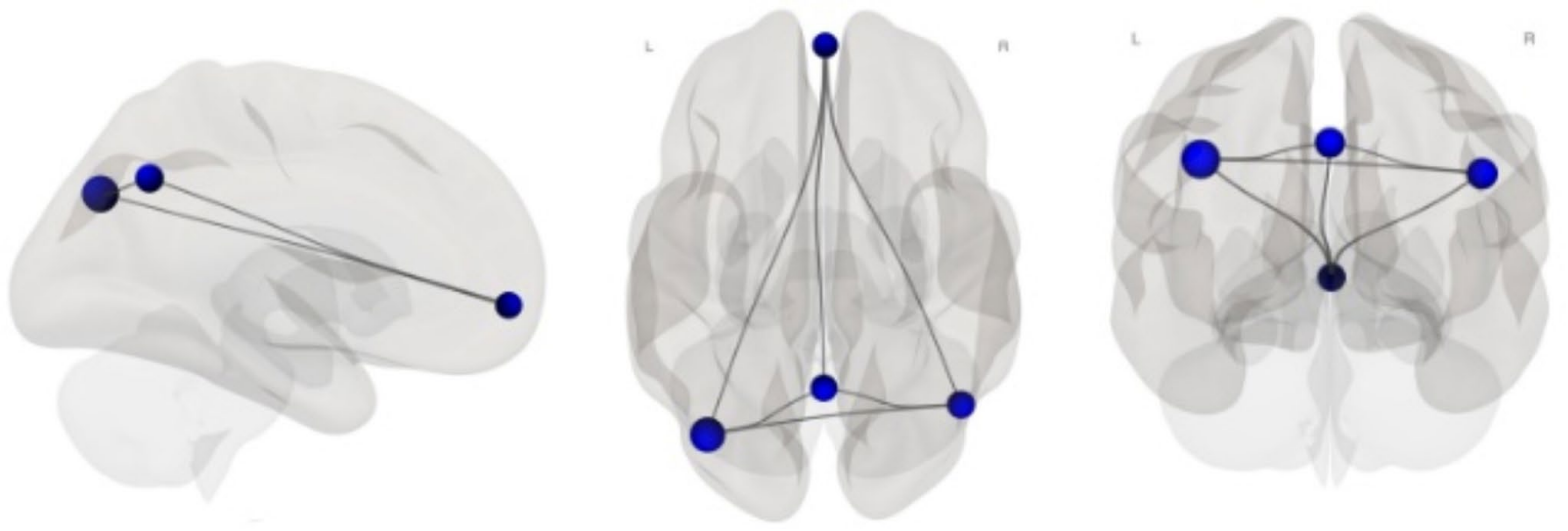
Global efficiency of default network as a function of VEGF-A levels. Note: The effect of VEGF-A levels on DMN connectivity is attributed to decreased efficiency between these nodes. Nodes are represented as blue circles, with strength of global efficiency represented by the size of the circle. Black lines represent edges between nodes

A total of 76 participants were included in the current analysis. Participant characteristics and vascular risk factors are reported in Table 1; nearly all of the participants in our sample who reported vascular risk factors were being treated for these conditions. Elevated levels of VEGF-A were associated with lower DMN connectivity [B = − 0.14, 95% CI (-0.26, − 0.01), *p* =.038], adjusting for age, sex, education and vascular risk factors (Fig. 1; Supplementary Table 1). The final model explained 12% of the observed variation in DMN connectivity [R^2^ = 0.12, F(5, 75) = 1.93, *p* =.100]. VEGF-A levels were associated with DMN connectivity even after adjusting for *APOE4* carrier status [B = − 0.13, 95% CI (-0.27, − 0.004), *p* =.044]. Graph theory analysis revealed that VEGF-A levels are associated with global efficiency of the entire network [B = − 0.18, *p* =.004], with decreased efficiency in the left [B = − 0.22, p-FDR = 0.011] and right [B = − 0.13, p-FDR = 0.020] lateral parietal cortex, followed by the precuneus cortex [B = − 0.17, p-FDR = 0.020] and medial prefrontal cortex [B = − 0.19, p-FDR = 0.021; Fig. 2]. Higher DMN connectivity was associated with higher age-adjusted MOANS scaled score on DRS, after accounting for sex and education [B = 4.06, 95% CI (0.72, 7.40), *p* =.018, *N* = 64].

**Table 1:** Participant characteristics, demographics and vascular risk factors

	All
	*N* = 76
Age (Years), M (SD)	70.3 (7.5)
Sex (Male), n (%)	24 (31.6)
Education (Years), M (SD)	16.5 (1.8)
*APOE4* Carrier, n (%)^1^	27 (37.0)
Vascular Risk Factors, n (%)	
Hypertension	30 (39.5)
Dyslipidemia^1^	41 (53.9)
Diabetes	6 (7.9)
Smoking History	26 (34.2)
TIA	1 (1.3)
Atrial Fibrillation^2^	2 (2.6)
Body Mass Index, M (SD)^3^	25.2 (4.8)
VEGF-A Level (pg/mL), M (SD)	103.6 (74.4)
DMN Connectivity (average correlation), M (SD)	0.48 (0.17)

*Note*: ^1^Missing for three participants; ^2^Missing for one participant; ^3^Missing for 22 participants

## Discussion

VEGF-A is a crucial angiogenic signaling protein involved in vascular homeostasis (Lange et al., 2016). However, it is also a potent permeability factor and the effect of VEGF-A on cognitive function and its role in neurocognitive and neurodegenerative conditions remains unknown (Dvorak et al., 1995). While prior studies have demonstrated both elevated and reduced levels of VEGF-A in individuals with cognitive impairment and dementia (Hohman et al., 2015; Tarkowski et al., 2002), no prior study has examined whether levels of VEGF-A may be associated with functional connectivity in the brain.

We observed that elevated levels of VEGF-A are associated with DMN connectivity, suggesting that elevated levels of VEGF-A may indicate a pathological or compensatory mechanism prior to the development of cognitive decline. Whether elevated levels of VEGF-A protect the cerebrovascular and cognitive function or contribute to the pathophysiological processes of neurocognitive conditions remains unclear.

Disruption of DMN connectivity often precedes the onset on cognitive decline (Hedden et al., 2009), and we observed an association between DMN connectivity and scores on a general cognitive status test. Prior studies have similarly reported decreased connectivity in DMN structures in the context of aging and dementia (Klaassens et al., 2017; Malagurski et al., 2022). Our findings that suggest elevated levels of VEGF-A are associated with such early pathological processes. Specifically, we observed that VEGF-A levels were associated with connectivity between the precuneus and left lateral parietal cortex. Prior studies have observed reduced oxygenation in the precuneus early in the progression of Alzheimer’s disease and reduced pericyte marker, platelet-derived growth factor receptor-β, within the precuneus and parietal white matter in Alzheimer’s disease (Miners et al., 2016, 2018). It is possible that hypoperfusion within these regions may be triggering angiogenic processes, indicated by elevated levels of VEGF-A. However, future studies are warranted to directly evaluate this hypothesis.

Limitations of the study include the small sample size and cross-sectional design. In addition, future studies could delineate the effect of vascular risk factors on angiogenic factors and whether angiogenic factors may moderate the association between vascular risk factors and functional connectivity as well as cognition. Moreover, participant demographics, such as level of education, may limit the generalizability of our results.

## Conclusion

This study is one of the first to examine whether VEGF-A may be linked to brain network connectivity metrics which precede cognitive decline. The findings of this study suggest that VEGF-A may be elevated early in the progression of neurocognitive disorders and highlight the need to evaluate the role of angiogenic and vascular mechanisms in the development of neurocognitive conditions. Additional larger longitudinal studies could inform the association between angiogenesis processes, functional connectivity breakdown and subsequent cognitive outcomes.

## Electronic supplementary material

Below is the link to the electronic supplementary material.

Supplementary Material 1

## Data Availability

The data that support the findings of this study are available from the corresponding author upon request.
