# Supplementary material for: Elevated vascular endothelial growth factor a is associated with disruption of default network connectivity in older adults
Source: Brain Imaging Behav. 2025 Feb 4;19(2):379–83. doi: 10.1007/s11682-025-00969-z (PMC11978527; doi:10.1007/s11682-025-00969-z)
Supplement: Supplementary file 1 — Supplementary Material 1 [file 11682_2025_969_MOESM1_ESM.docx]

**Supplementary Material**

**Neuroimaging Scan Parameters**

All participants underwent brain MRI on a 3T scanner (Siemens MAGNETOM Prisma System). The following sequences were examined for the current analysis: 3D T1-weighted MPRAGE anatomical scan for qualitative assessment of brain structures and abnormalities (scan parameters: TR = 2300 ms; TE = 2.98 ms; TI = 900 ms; flip angle = 9 deg; FOV = 256 mm; resolution = 1.0 × 1.0 × 1.2 mm^3^; scan time = 9 minutes), and resting state fMRI (rsfMRI) to determine blood-oxygen-level-dependent (BOLD) signal (scan parameters: TR = 3000 ms; TE = 30 ms; flip angle = 80 deg; FOV = 212 mm; voxel size = 3.3 × 3.3 × 3.3 mm; matrix = 64 × 64; number of slices = 48, slice order = interleaved; number of time points (scans) = 140 contiguous echo-planar imaging (EPI); scan time = 7 min 11 s).

**Supplementary Table 1. Association Between VEGF-A Levels and DMN Connectivity in Multiple Regression**

| Variable | Unstandardized Coefficients | | Standardized Coefficients | t | Sig. | 95% Confidence Interval for B | |
| --- | --- | --- | --- | --- | --- | --- | --- |
|  | B | Std. Error |  |  |  | Lower Bound | Upper Bound |
| **Age** (Years) | 0.003 | 0.003 | 0.139 | 1.21 | .229 | -0.002 | 0.008 |
| **Sex** (Male) | -0.062 | 0.042 | -0.173 | -1.49 | .142 | -0.145 | 0.021 |
| **Education** (Years) | -0.009 | 0.011 | -0.097 | -0.84 | .406 | -0.031 | 0.013 |
| **Vascular Risk Factors**  (0-1 vs. ≥ 2) | 0.043 | 0.039 | 0.126 | 1.09 | .280 | -0.035 | 0.121 |
| **VEGF-A Levels** (pg/mL; log-transformed) | -0.136 | 0.064 | -0.242 | -2.12 | .038 | -0.264 | -0.008 |

*Dependent Variable: DMN connectivity (average Fisher's z-transformed correlation)*

**Supplemental Figure 1. Participant Inclusion Flow Chart**

Available for Analysis

**N = 77**

Incidental Finding on MRI

**n = 9**

VEGF-A Outlier

**n = 1**

Included in Analysis

**N = 76**

Cardiovascular Disease

**Present: n = 7**

**Missing Data: n = 2**

**Eligible Participants***

**n = 95**

*Participants with valid BOLD scans and available VEGF-A levels.
